# Supplementary material for: Photoperiodic Flowering Response of Essential Oil, Grain, and Fiber Hemp (Cannabis sativa L.) Cultivars
Source: Front Plant Sci. 2021 Aug 2;12:694153. doi: 10.3389/fpls.2021.694153 (PMC8367441; doi:10.3389/fpls.2021.694153)
Supplement: Supplementary file 1 [file Data_Sheet_1.docx]

Supplementary Material

# Supplementary table 1. Germplasm source, type, and referred name for fifteen essential oil hemp cultivars.

| Cultivar | Source | Type | Referred Name |
| --- | --- | --- | --- |
| ACDC | A | Cuttings | ACDC-AC |
| Super CBD | A | Cuttings | Super CBD-AC |
| Cherry | A | Cuttings | Cherry-AC |
| Wife | A | Cuttings | Wife-AC |
| Cherry Blossom | B | Cuttings | Cherry Blossom-BC |
| Cherry Wine | B | Seeds | Cherry Wine-BS |
| Cherry Blossom | B | Seeds | Cherry Blossom-BS |
| Cherry*T1 | B | Seeds | Cherry*T1-BS |
| Berry Blossom | B | Seeds | Berry Blossom-BS |
| Cherry Blossom-Tuan | B | Seeds | Cherry Blossom-Tuan-BS |
| JL Baux | C | Cuttings | JL Baux-CC |
| ACDC | C | Cuttings | ACDC-CC |
| Cherry Wine | C | Cuttings | Cherry Wine-CC |
| Cherry | C | Cuttings | Cherry-CC |
| Wife | C | Cuttings | Wife-CC |

# Supplementary table 2. Means (± SE) of controlled rooms air temperature, relative humidity, and photosynthetic photon flux density (PPFD) of photoperiod treatments for essential oil, fiber, and grain cultivars in Expt. 1, 2, 3, and 4 as measured by thermocouples and quantum sensors. Light intensity was measure at ten representative positions at plant canopy level at the onset of each growing stage while air temperature and relative humidity were recorded by a wireless data logger throughout the growing stages.

| Treatment | Vegetative Stage | | | Flowering Stage | | |
| --- | --- | --- | --- | --- | --- | --- |
|  | Air temperature (℃) | Relative humidity (%) | PPFD  (µmol·m^-2^·s^-1^) | Air temperature (℃) | Relative humidity (%) | PPFD  (µmol·m^-2^·s^-1^) |
|  | Expt. 1 | | | | | |
| 12h | 25.3 ± 0.02 | 57.4 ± 0.14 | 287.0 ± 12.80 | 24.2 ± 0.01 | 84.8 ± 0.09 | 346.3 ± 11.54 |
| 12h30min | 24.7 ± 0.02 | 51.4 ± 0.13 | 289.4 ± 10.49 | 24.2 ± 0.01 | 79.4 ± 0.07 | 331.1 ± 8.67 |
| 13h | 23.9 ± 0.03 | 58.5 ± 0.15 | 309.8 ± 12.91 | 24.3 ± 0.02 | 80.8 ± 0.09 | 336.3 ± 12.81 |
| 13h30min | 26.0 ± 0.03 | 59.7 ± 0.16 | 299.1 ± 10.03 | 24.3 ± 0.02 | 81.6 ± 0.09 | 322.1 ± 10.11 |
| 13h45min | 24.5 ± 0.02 | 56.6 ± 0.14 | 309.3 ± 7.09 | 24.4 ± 0.02 | 83.4 ± 0.07 | 334.1 ± 12.36 |
| 14h | 24.8 ± 0.04 | 55.1 ± 0.16 | 296.1 ± 10.56 | 24.5 ± 0.03 | 80.2 ± 0.08 | 333.5 ± 13.82 |
| 18h | 25.0 ± 0.02 | 57.3 ± 0.12 | 294.0 ± 9.77 | 24.4 ± 0.01 | 80.2 ± 0.07 | 330.0 ± 13.82 |
|  | Expt. 2 | | | | | |
| 12h | 25.2 ± 0.02 | 56.6 ± 0.16 | 288.6 ± 10.27 | 24.8 ± 0.02 | 73.8 ± 0.08 | 326.4 ± 11.56 |
| 13h30min | 25.8 ± 0.05 | 50.9 ± 0.17 | 305.3 ± 10.41 | 24.6 ± 0.03 | 70.7 ± 0.09 | 344.2 ± 9.76 |
| 13h45min | 26.6 ± 0.05 | 48.5 ± 0.15 | 305.7 ± 8.16 | 24. 6 ± 0.03 | 66.3 ± 0.08 | 348.7 ± 11.85 |
| 14h | 23.3 ± 0.10 | 47.2 ± 0.13 | 302.3 ± 7.39 | 25.1 ± 0.04 | 72.0 ± 0.10 | 343.5 ± 9.14 |
| 14h30min | 25.8 ± 0.03 | 52.5 ± 0.16 | 305.7 ± 12.31 | 24.5 ± 0.01 | 67.6 ± 0.09 | 347.0 ± 8.70 |
| 14h45min | 25.4 ± 0.02 | 48.9 ± 0.14 | 292.5 ± 8.97 | 24.5 ± 0.01 | 62.3 ± 0.07 | 338.9 ± 7.74 |
| 18h | 25.7 ± 0.03 | 50.6 ± 0.17 | 280.5 ± 8.17 | 24.4 ± 0.01 | 61.4 ± 0.09 | 329.6 ± 11.97 |
|  | Expt. 3 | | | | | |
| 12h30min | 27.0 ± 0.06 | 83.8 ± 0.18 | 271.6 ± 5.44 | 25.1 ± 0.02 | 48.7 ± 0.09 | 345.9 ± 7.37 |
| 13h |  |  |  | 25.1 ± 0.01 | 43.5 ± 0.07 | 341 ± 10.17 |
| 14h30min |  |  |  | 24.9 ± 0.02 | 51.4 ± 0.09 | 337.2 ± 10.60 |
| 14h45min |  |  |  | 25.7 ± 0.02 | 53.1 ± 0.07 | 321.4 ± 9.59 |
| 15h |  |  |  | 26.4 ± 0.05 | 49.9 ± 0.08 | 321.6 ± 15.84 |
| 15h30min |  |  |  | 25.0 ± 0.02 | 52.4 ± 0.09 | 329.0 ± 8.77 |
|  | Expt. 4 | | | | | |
| Natural day length | 31.7± 0.19 | 56.0 ± 0.40 | 250.9 ± 6.19 | 27.3 ± 0.03* | 83.4 ± 0.13* | 221.6 ± 2.85*  (W·m^-2^) |

*Data was recorded by Florida Automated Weather Network with a pyranometer measuring 400 to 1100 nm radiation.


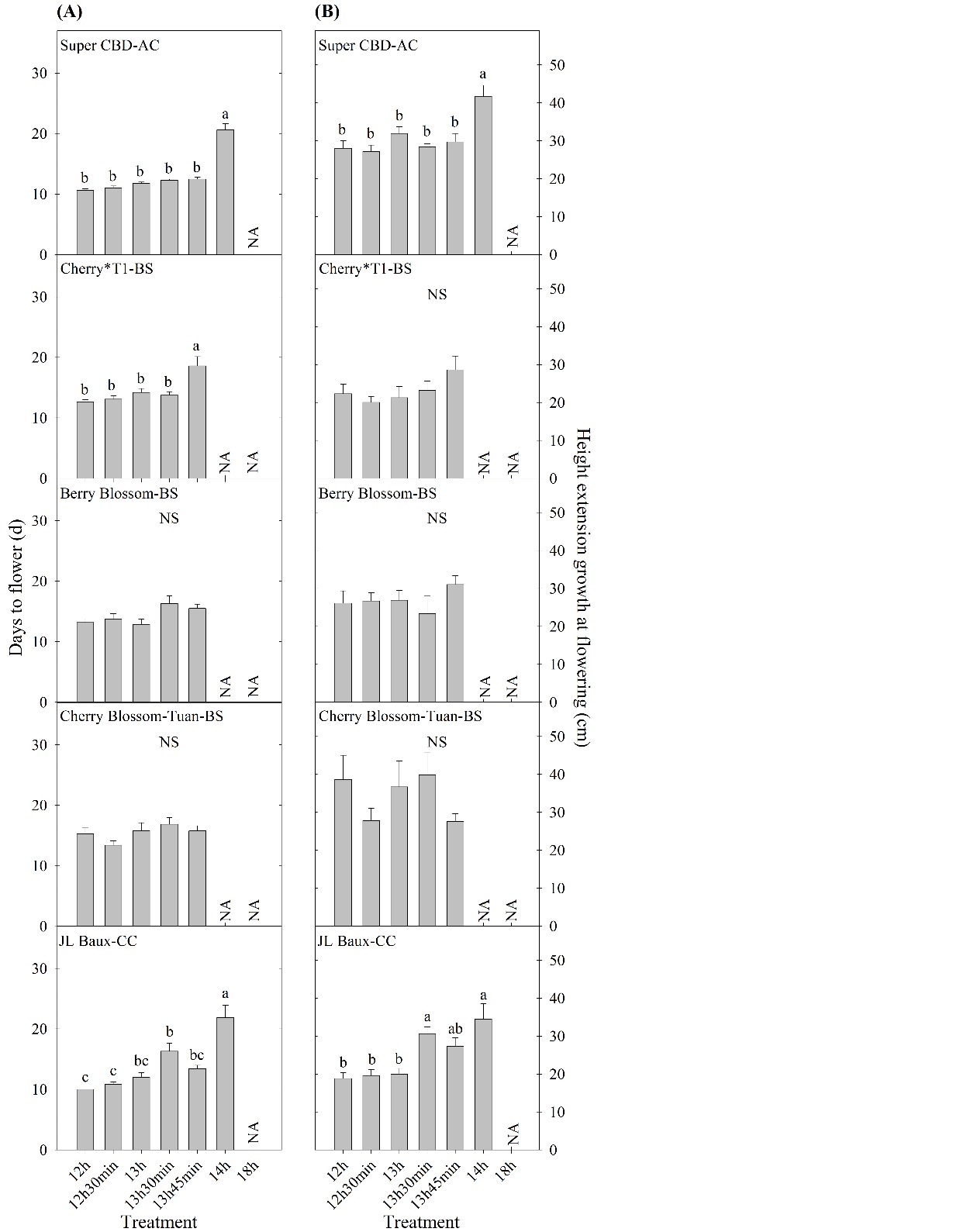


Supplementary figure 1. Days to flower (A) and height extension growth at flowering (B) of five essential oil cultivars in Expt. 1. All data were pooled from ten replications except ‘JL Baux-CC’ (eight replications). NS indicates insignificant treatment effects. NA indicates the majority of the 10 replicates (i.e. 6/10) were reported as not flowering. Means sharing a letter are not statistically different by Tukey’s honestly significant difference test at P ≤ 0.05. Error bars indicate standard error.


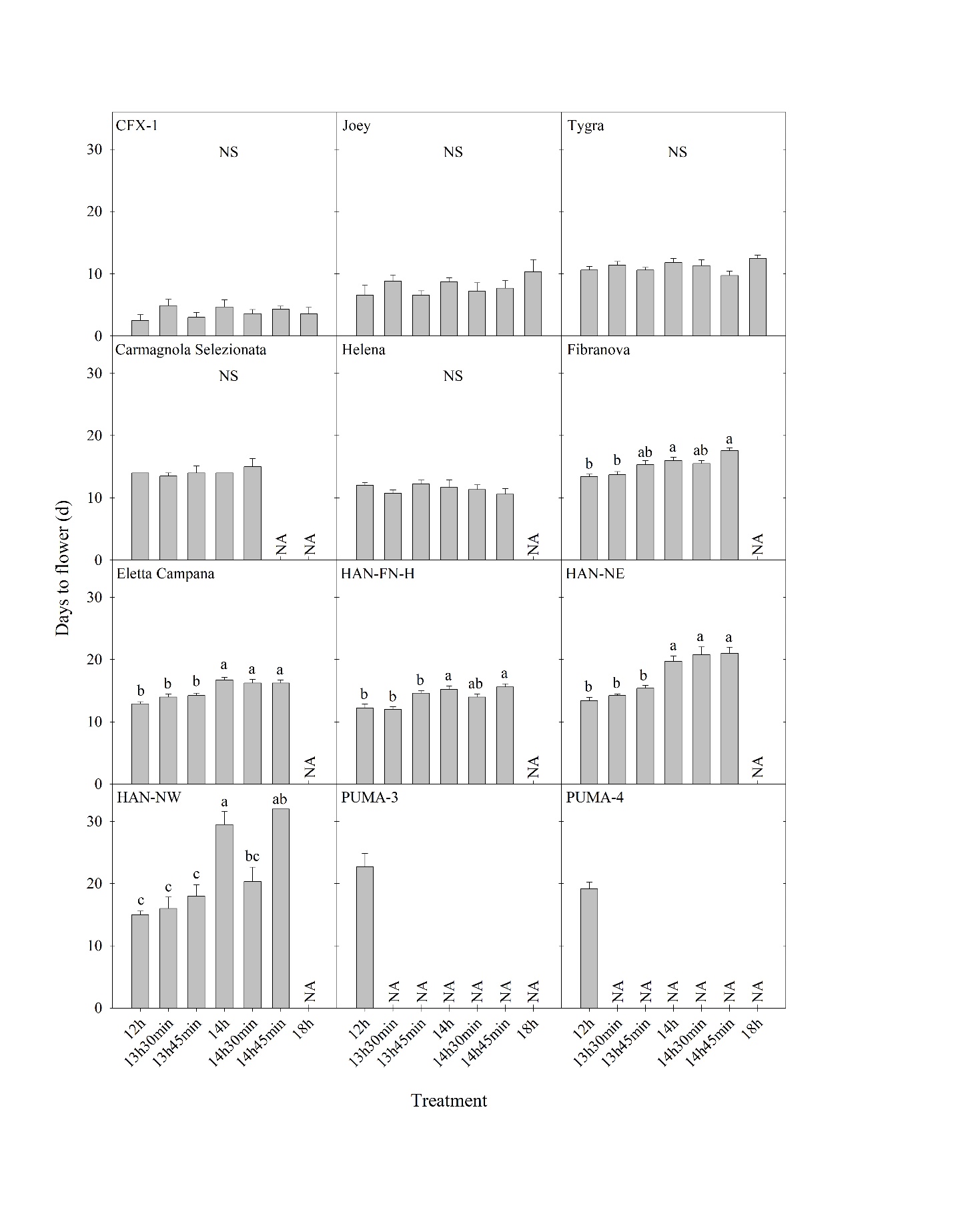


**Supplementary figure 2.** Days to flower of twelve fiber/grain hemp cultivars in Expt. 3. All data were pooled from multiple replications as described in the Methods. NS indicates insignificant treatment effects. NA indicates the less than three plants were reported as not flowering. Means sharing a letter are not statistically different by Tukey’s honestly significant difference test at P ≤ 0.05. Error bars indicate standard error.


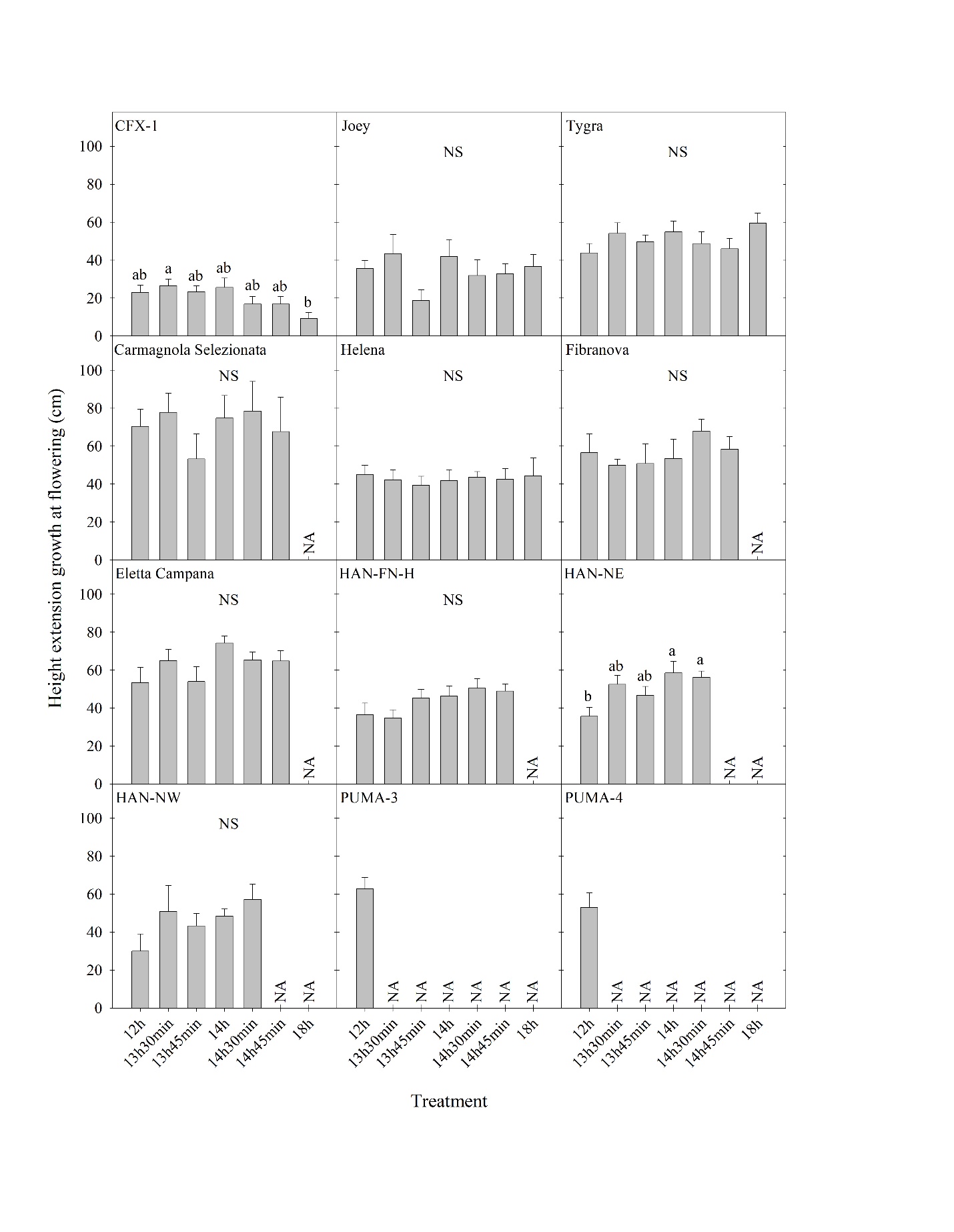


**Supplementary figure 3.** Height extension growth at flowering of twelve fiber/grain hemp cultivars in Expt. 3. All data were pooled from multiple replications as described in the Methods. NS indicates insignificant treatment effects. NA indicates the less than three plants were reported as not flowering. Means sharing a letter are not statistically different by Tukey’s honestly significant difference test at P ≤ 0.05. Error bars indicate standard error.
